# Resuscitative endovascular balloon occlusion of the aorta as an adjunct for hemorrhagic shock due to uterine rupture: a case report

**DOI:** 10.1002/ccr3.1126

**Published:** 2017-08-15

**Authors:** Asami Okada, Osamu Nakamoto, Maya Komori, Hideki Arimoto, Hiroshi Rinka, Hiroaki Nakamura

**Affiliations:** ^1^ Department of Obstetrics and Gynecology Osaka City General Hospital 2‐13‐22 Miyakojimahondori Miyakojima‐ku Osaka Japan; ^2^ Department of Emergency and Critical Care Medical Center Osaka City General Hospital 2‐13‐22 Miyakojimahondori Miyakojima‐ku Osaka Japan

**Keywords:** Intra‐aortic balloon occlusion, late pregnancy, perinatal period, placenta accreta, resuscitative endovascular balloon occlusion of the aorta

## Abstract

Resuscitative endovascular balloon occlusion of the aorta (REBOA) is a life‐saving procedure used to control bleeding and maintain blood pressure temporarily in traumatic hemorrhagic shock. Uterine rupture and placenta accreta provoke uncontrollable massive hemorrhaging. REBOA may be useful for hemodynamic stabilization to prevent cardiac arrest in high‐risk pregnancy.

## Introduction

Hemorrhagic shock is an emergency associated with high mortality, occurring not only in trauma but also in the perinatal period. It is the most common cause of maternal death in Japan 1. Immediate control of bleeding and large‐volume blood transfusion are critical for patient survival. Resuscitative endovascular balloon occlusion of the aorta (REBOA) is a life‐saving procedure used to control bleeding and maintain blood pressure temporarily in traumatic hemorrhagic shock 2. This simple procedure involves obtaining femoral artery access, passing a wire into the aorta, and placing an occlusion balloon at the appropriate aortic position. Implementation of REBOA is easier and faster than implementation of other balloon occlusion strategies, such as common iliac artery balloon occlusion, which needs transfer to angiography room used in the perinatal period, and is less invasive than aortic cross‐clamping of resuscitative thoracotomy. In addition, hemorrhagic shock is a challenging situation in the perinatal period. There are some studies that support the use of prophylactic balloon occlusion of the aorta or iliac artery to control bleeding in cases of placenta accreta 3, 4; however, to the best of our knowledge, there are no reports on REBOA for the treatment of hemorrhagic shock in late pregnancy. Here, we describe a case wherein REBOA was used for the treatment of life‐threatening hemorrhagic shock in late pregnancy due to uterine rupture.

## Case Examination

A 40‐year‐old Japanese woman was admitted to our Obstetrics and Gynecology Department for the management of placenta accreta at 30 weeks of gestation. We predicted massive bleeding during cesarean section; therefore, we planned to use an iliac artery balloon occlusion strategy during the cesarean section. At 35 weeks of gestation, she suddenly collapsed in her ward. She was in shock with a blood pressure of 54/38 mmHg, a heart rate of 99 bpm, a respiratory rate of 32 breaths/min, and a peripheral oxygen saturation of 99% (room air). She complained of abdominal pain, and her abdomen was distended. Laboratory data revealed decreased hemoglobin (7.1 g/dL) compared to 1 h previously (9.8 g/dL). Therefore, we suspected intra‐abdominal bleeding. Because her vital signs did not improve with fluid resuscitation, we decided to perform emergency cesarean section immediately.

**Figure 1:**
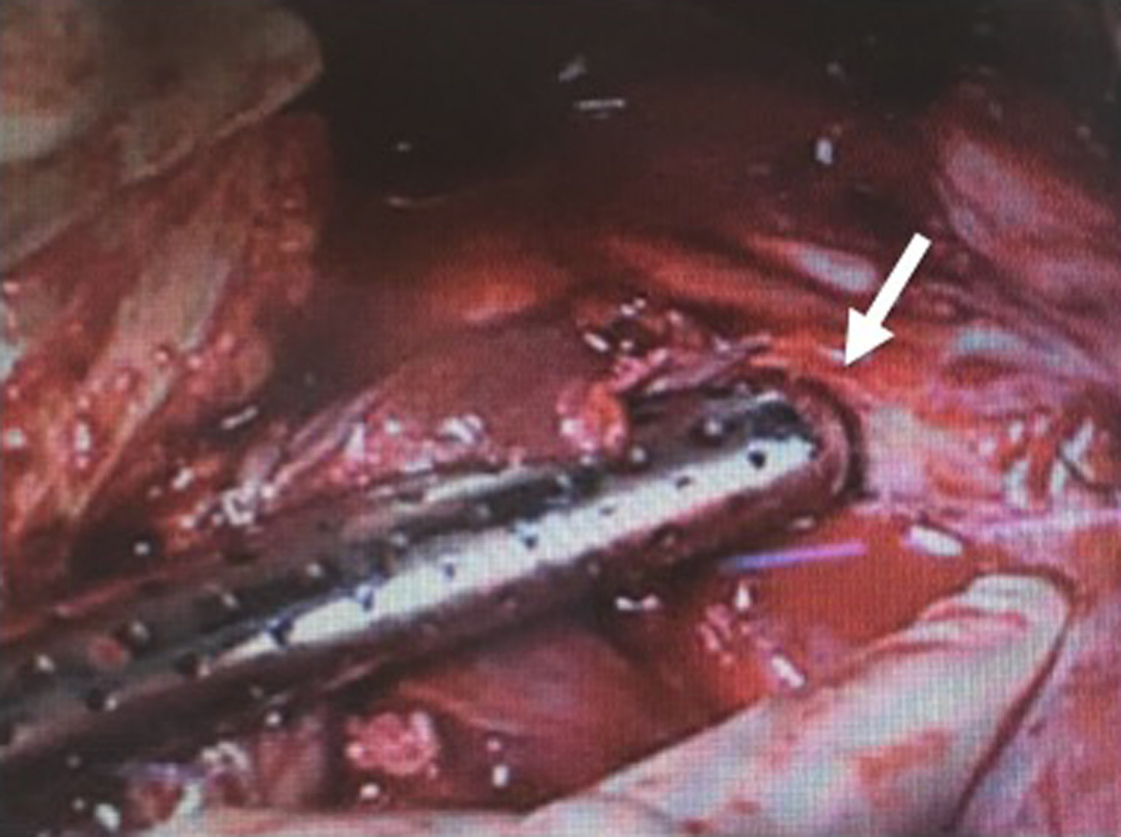
Photograph of uterine rupture. Massive intra‐abdominal bleeding was caused by a 1‐cm laceration of the uterine wall (arrow).

The patient was transported to the operating theater 10 min later. We predicted life‐threatening hemorrhage because of placenta accreta and decided to implement REBOA during preparation for surgery in order to prevent cardiac arrest. The acute care surgeon accessed the right femoral artery for REBOA using a 7‐French sheath (Rescue balloon^®^, Tokai Medical Products, Japan) by the Seldinger method at blindly and placed the balloon above the bifurcation of the aorta using guided fluoroscopy in the operating theater for 7 min during initiation of intubation and general anesthesia. Balloon occlusion immediately improved the systemic blood pressure from 50 mmHg to 120 mmHg. Surgery was immediately started (36 min from the initial decision). Massive intra‐abdominal bleeding caused by an approximately 1‐cm uterine laceration was found, and uterine rupture was diagnosed (Fig. 1). The infant was delivered 4 min after balloon inflation. A hysterectomy was performed after delivery of the infant. REBOA was performed intermittently during the surgery, and blood pressure was maintained. The total duration of occlusion was 54 min (divided into 29 min and 25 min, both were total occlusion). We got clear and dry surgical field during balloon inflation; however, we lost that after bleeding when the balloon deflated. The total operation time was approximately 180 min, and the amount of bleeding was 6970 g (Table 1). Packed red blood cells (20 units), fresh‐frozen plasma (20 units), and platelets (20 units) were administered.

**Table 1:** Time course

Time	
00:00	Decided to perform emergency operation
00:10	Enter the operating theater
00:26	REBOA insertion
00:33	REVOA total inflate
00:35	Surgery was started
00:41	The delivery of infant
01:02	REBOA deflate
01:07	REBOA inflate
01:32	REBOA deflate
01:34	Hysterectomy was performed
03:36	Completion of surgery

On admission to the intensive care unit, the patient was hemodynamically stable. At postoperative day 1, she was transferred to the obstetrics ward and subsequently discharged on day 14 without complications. The baby weighed 1990 g at birth. Apgar scores of 1 (1 min) and 5 (5 min) were recorded. The umbilical artery blood gas analysis of the baby was below PH 6.981 with a base deficit of −15.6 mmol/L indicating metabolic acidosis. The baby's condition gradually improved, and she was discharged uneventfully on day 23.

## Discussion

Uterine rupture is a rare but severe condition that can cause severe hemorrhagic shock and is associated with a high mortality rate 5. Moreover, placenta accreta is a common cause of maternal death and provokes uncontrollable massive hemorrhaging 6, 7.

Here, we describe a case of life‐threatening hemorrhagic shock due to uterine rupture with placenta accreta in late pregnancy that was successfully treated using REBOA.

There were two key observations in this case. First, REBOA could prevent cardiac arrest even during late pregnancy. Currently, REBOA is used as a common and effective procedure for hemodynamic support in trauma 8 and is useful for managing postpartum hemorrhagic shock 9. Moreover, prophylactic aortic balloon occlusion for placenta accreta in cesarean section has been reported as a useful procedure to reduce the amount of bleeding 4, 10.

On the other hand, delay to definitive therapy is the most important disadvantage of REBOA. Inoue et al. 11 indicated that delay in definitive hemostasis after REBOA might be one of its drawbacks and may result in high mortality. We agree with this and in our case, we decided to perform definitive surgery immediately and transferred the patient to the operating theater as soon as possible. We predicted that hemorrhage because of placenta accreta would be very difficult to control with the potential of cardiac arrest. We also predicted that implementation of REBOA would not delay definitive surgery. REBOA was performed in only 7 min during preparation for the operation, and it successfully prevented cardiac arrest and maintained hemodynamic status. The average time from initiation to successful REBOA in the emergency department or operating theater has been reported to be 6.6 min 12. The time for induction of REBOA in our case was the same as that of reported in severe trauma cases. Therefore, REBOA did not cause delay in hemostasis.

Second, REBOA provided a clear view of surgical field without bleeding. When the REBOA was deflated during the emergency cesarean section and hysterectomy, we could not obtain a surgical field or continue the operation because of bleeding. However, after REBOA was performed again, the bleeding decreased and we were able to maintain a dry surgical field. Therefore, REBOA enabled us to perform surgery smoothly in a case of intra‐abdominal massive bleeding. There are some reports that REBOA decreases bleeding 4, 10; therefore, we believe that REBOA provides a reasonably dry field.

There are some important considerations for the use of REBOA over other balloon occlusion strategies in the perinatal period. First, it may be more difficult to obtain femoral artery access compared to postpartum patients or nonpregnant patients because the artery may not be palpable because of hypotension and spasm in severe shock. In addition, abdominal distension in late pregnancy does not allow sufficient working space for easy femoral artery access. Moreover, the uterus may press against the external and common iliac artery in late pregnancy. Furthermore, the time to implement REBOA may cause a delay in initiation of definitive treatment. Therefore, cooperation of acute care surgeons or well‐experienced interventionists is necessary. Modification of REBOA using sonography, fluoroscopy, or a cut‐down procedure may improve immediate access to the femoral artery and allow its more widespread use in late pregnancy.

The second consideration when performing REBOA in late pregnancy is the potential effect on the fetus because the fetal safety profile for REBOA during pregnancy is unknown. However, in a situation of impending cardiac arrest, hemodynamic stabilization of the mother takes priority over potential risks to the fetus. There are several reports on preoperative balloon occlusion in cesarean section to control massive bleeding. Wu et al. reported that there was no significant difference in the Apgar scores of the neonates born after cesarean section who underwent prophylactic balloon occlusion and who did not undergo balloon occlusion. Further, about the fetal radiation exposure, they concluded that the radiation exposure for prophylactic balloon occlusion is mostly safe for the fetus 13. In addition, Chen et al. reported that the balloon occlusion of aorta seems effective in reducing postpartum hemorrhage and in enabling blood transfusion without harming the newborns 14. In our institution, we performed 14 preoperative balloon occlusions in cesarean section (12 common iliac artery balloon occlusions and two aortic balloon occlusions) with monitoring of the cardiotocogram of the fetus during the inflation. In these cases, the time from balloon inflation to delivery of the fetus was within 5 min, and there was no adverse effect on babies. The time in this case was also within 5 min. Therefore, we considered that REBOA did not cause any adverse effects and that the metabolic acidosis of the baby was provoked by the hemorrhagic shock due to uterine rupture.

Third, REBOA risks ischemic complications 11, 15 and we bore this in mind.

Accordingly, the indication for REBOA should always be considered. However, it may be useful to support hemodynamic state and obtain dry surgical field in life‐threatening hemorrhage due to uterine rupture.

## Conclusions

We describe the use of REBOA for life‐threatening hemorrhagic shock due to uterine rupture. REBOA may be useful for maternal hemodynamic support, to prevent cardiac arrest and to obtain a dry surgical field. However, REBOA carries potential risks, and therefore, its indication should be considered.

## Authorship

AO: drafted the manuscript. ON and HN: discussed during patient care and prepared the manuscript. HA and HR: contributed to operative and postoperative cares.

## Competing Interests

The authors declare that they have no competing interests.

## References

[1] Hasegawa, J. , T. Ikeda , A. Sekizawa , H. Tanaka , M. Nakamura , S. Katsuragi , et al. 2016 Recommendations for saving mothers’ lives in Japan: report from the Maternal Death Exploratory Committee (2010‐2014). J. Obstet. Gynecol. Res. 42:1637–1643. 27718278 10.1111/jog.13136

[2] Morrison, J. J. , T. J. Percival , N. P. Markov , C. Villamaria , D. J. Scott , K. A. Saches , et al. 2012 Aortic balloon occlusion is effective in controlling pelvic hemorrhage. J. Surg. Res. 177:341–347. 22591921 10.1016/j.jss.2012.04.035

[3] Panici, P. B. , M. Anceschi , M. L. Borgia , L. Bresadola , G. Masselli , T. Parasassi , et al. 2012 Intraoperative aorta balloon occlusion: fertility preservation in patients with placenta previa accreta/increta. J. Matern. Fetal Neonatal Med. 25:2512–2516. 22992070 10.3109/14767058.2012.712566

[4] Matsuoka, K. , T. Kawabata , and K. Yoza . 2015 Anesthetic management of patients with placenta previa accreta for cesarean section: a 7‐year single‐center experience. Masui 64:70–76. 25868205

[5] Barger, M. K. , A. Nannini , S. DeJoy , K. Wisner , and G. Markenson . 2013 Maternal and newborn outcomes following uterine rupture among women without versus those with a prior cesarean. J. Matern. Fetal Neonatal Med. 26:183–187. 22954425 10.3109/14767058.2012.725790

[6] Breen, J. L. , R. Neubecker , C. A. Gregori , and J. E. Jr Franklin . 1977 Placenta accreta, increta, and percreta. A survey of 40 cases. Obstet. Gynecol. 49:43–47. 299782

[7] Knight, M. , C. Acosta , P. Brocklehurst , A. Cheshire , K. Fitzpatrick , L. Hinton , et al. 2016 Beyond maternal death: improving the quality of maternal care through national studies of ‘near‐miss’ maternal morbidity. Programme Grants Appl. Res., Southampton (UK). 27386616

[8] Morrison, J. J. , R. E. Galgon , J. O. Jansen , J. W. Cannon , T. E. Rasmussen , and J. L. Eliason . 2016 A systematic review of the use of resuscitative endovascular balloon occlusion of the aorta in the management of hemorrhagic shock. J. Trauma Acute Care Surg. 80:324–334. 26816219 10.1097/TA.0000000000000913

[9] Harma, M. , M. Harma , A. S. Kunt , M. H. Andac , and N. Demir . 2004 Balloon occlusion of the descending aorta in the treatment of severe post‐partum haemorrhage. Aust. N. Z. J. Obstet. Gynaecol. 44:170–171. 15089847 10.1111/j.1479-828X.2004.00181.x

[10] Noguchi, N. , S. Izumi , K. Kamizato , S. Nakamura , M. Kakinohana , and K. Sugahara . 2014 Anesthetic management of intra‐aortic balloon occlusion (IABO) for seven cases of placenta accreta–a six year experience at our institute. Masui 63:1334–1338. 25669086

[11] Inoue, J. , A. Shiraishi , A. Yoshiyuki , K. Haruta , H. Matsui , and Y. Otomo . 2016 Resuscitative endovascular balloon occlusion of the aorta might be dangerous in patients with severe torso trauma: a propensity score analysis. J. Trauma Acute Care Surg. 80:559–566. discussion 66‐7. 26808039 10.1097/TA.0000000000000968

[12] DuBose, J. J. , T. M. Scalea , M. Brenner , D. Skiada , K. Inaba , J. Cannon , et al. 2016 The AAST prospective Aortic Occlusion for Resuscitation in Trauma and Acute Care Surgery (AORTA) registry: data on contemporary utilization and outcomes of aortic occlusion and resuscitative balloon occlusion of the aorta (REBOA). J. Trauma Acute Care Surg. 81:409–419. 27050883 10.1097/TA.0000000000001079

[13] Wu, Q. , Z. Liu , X. Zhao , C. Liu , Y. Wang , Q. Chu , et al. 2016 Outcome of pregnancies after balloon occlusion of the infrarenal abdominal aorta during caesarean in 230 patients with placenta praevia accreta. Cardiovasc. Intervent. Radiol. 39:1573–1579. 27439624 10.1007/s00270-016-1418-y PMC5052309

[14] Chen, M. , and L. Xie . 2016 Clinical evaluation of balloon occlusion of the lower abdominal aorta in patients with placenta previa and previous cesarean section: a retrospective study on 43 cases. Int. J. Surg. 34:6–9. 27545958 10.1016/j.ijsu.2016.08.016

[15] Okada, Y. , H. Narumiya , W. Ishi , and I. Ryoji . 2016 Lower limb ischemia caused by resuscitative balloon occlusion of aorta. Surg. Case Rep. 2:130. 27834057 10.1186/s40792-016-0260-4 PMC5104701

